# Clinical retrospective study on serum sCD163 and CXCL10 in the immune microenvironment of carotid atherosclerosis: association with inflammatory cytokines, immune cell subsets, and disease progression

**DOI:** 10.3389/fimmu.2026.1834236

**Published:** 2026-05-07

**Authors:** Hong Pan, Huijun Zheng, Mi Li

**Affiliations:** 1 Department of Ultrasound Medicine, Wenzhou Traditional Chinese Medicine (TCM) Hospital of Zhejiang Chinese Medical University, Wenzhou, Zhejiang, China; 2 Department of Traditional Chinese Medicine Gynecology, Wenzhou Traditional Chinese Medicine (TCM) Hospital of Zhejiang Chinese Medical University, Wenzhou, Zhejiang, China; 3 Rehabilitation Department, Wenzhou Traditional Chinese Medicine (TCM) Hospital of Zhejiang Chinese Medical University, Wenzhou, Zhejiang, China

**Keywords:** carotid atherosclerosis, CXCL10, disease progression, immune microenvironment, inflammatory cytokines, predictive biomarkers, radiological progression, sCD163

## Abstract

**Background:**

Carotid atherosclerosis progression involves complex immune-inflammatory responses, but the interplay within its microenvironment between the macrophage marker sCD163 and the Th1/NK cell-recruiting chemokine CXCL10 remains unclear.

**Objective:**

To investigate the correlations between serum levels of sCD163 and CXCL10 and the profiles of inflammatory cytokines, immune cell subsets, and 12-month radiological progression in patients with carotid atherosclerosis.

**Methods:**

We enrolled 82 patients with carotid atherosclerosis from January 2022 to December 2024, along with 82 healthy controls from concurrent physical examinations. All patients had undergone baseline carotid ultrasound and a subsequent ultrasound after 12 months as part of routine clinical care. Based on these 12−month imaging results, patients were retrospectively categorized as having stable disease (n=42) or radiological progression (n=40). Clinical data were collected for all subjects, and serum levels of sCD163, CXCL10, and inflammatory cytokines were measured by ELISA. Flow cytometry was used to analyze peripheral blood lymphocyte subsets. Correlations between sCD163, CXCL10, cytokines, and immune cell subsets were assessed using Pearson or Spearman analysis. The predictive value of sCD163 and CXCL10 for disease progression was evaluated by ROC curve analysis.

**Results:**

The radiological progression group had significantly higher serum levels of sCD163, CXCL10, and inflammatory cytokines (IL-6, IL-1β, TNF-α) (all *P* < 0.001) compared to both the healthy control and disease-stable groups. Immune cell analysis also showed a lower CD8^+^ T cell percentage and a higher CD4^+^/CD8^+^ ratio in the progression group (both *P* < 0.001). Correlation analysis indicated that sCD163 and CXCL10 were both positively correlated with inflammatory cytokine levels and the CD4^+^/CD8^+^ ratio, and negatively correlated with the percentage of CD8^+^ T cells (all *P* < 0.001). ROC curve analysis showed that the AUC for baseline serum CXCL10 in predicting radiological progression was 0.875 (95% CI: 0.783-0.938), and for sCD163 was 0.767 (95% CI: 0.660-0.853). The combined prediction using both markers increased the AUC to 0.913 (95% CI: 0.830-0.964).

**Conclusion:**

Serum levels of sCD163 and CXCL10 are closely associated with systemic inflammatory status, immune cell imbalance, and radiological progression in patients with carotid atherosclerosis. The combined detection of these two markers demonstrates good predictive efficacy for disease progression.

## 1 Introduction

Carotid atherosclerotic plaques constitute a critical pathological basis for the occurrence of ischemic stroke. The primary mechanisms involve embolism arising from the rupture of vulnerable plaques and hemodynamic impairment in the intracranial circulation caused by severe luminal stenosis (1, 2). Studies indicate that approximately 18% to 25% of ischemic strokes are directly associated with the rupture of carotid atherosclerotic plaques, events that are often accompanied by a high mortality risk (3). Atherosclerosis is fundamentally a chronic immuno-inflammatory disease characterized by plaque formation within the arterial wall, leading to luminal stenosis or even occlusion, and consequently, insufficient blood supply to target organs (4, 5). Therefore, a deeper analysis of the features of the local immune microenvironment in carotid atherosclerosis, as well as systemic immuno-inflammatory markers, holds paramount scientific and clinical significance for elucidating disease mechanisms, identifying high-risk patients, and developing novel intervention strategies.

Atherosclerotic plaques are far from being static lipid deposits; they represent a highly active immune microenvironment rich in dynamic intercellular interactions (6). In this complex process, the monocyte-macrophage system plays a central role. Circulating monocytes infiltrate into the subendothelial space under the influence of chemotactic factors, differentiate into macrophages, and then extensively uptake oxidized low-density lipoprotein via scavenger receptors, transforming into foam cells that form the lipid core of the plaque (7, 8). CD163 is preferentially expressed on M2 and tissue-resident macrophages (9). Its soluble form (sCD163) is released into the bloodstream after proteolytic cleavage and holds diagnostic and prognostic value in chronic inflammation, tissue repair, and various diseases (10).

In addition to the innate immune system, adaptive immune responses, particularly those mediated by T lymphocytes, are also indispensable in the regulation of atherosclerosis (11–13). The T cells infiltrating the plaque are predominantly CD4^+^ T helper cells, among which Th1 cells strongly promote pro-inflammatory responses by secreting cytokines such as interferon-γ (IFN-γ). These cytokines activate macrophages toward M1 polarization while inhibiting M2 differentiation (14). Chemokines play a crucial role in directing specific immune cells to the site of inflammation. CXCL10 is a representative member. It is mainly produced by IFN-γ-stimulated endothelial cells, macrophages, and other cells, and specifically recruits Th1 cells, cytotoxic T cells, and NK cells expressing its receptor CXCR3 to the inflammatory site through binding to CXCR3 (15). Previous studies have established that circulating CXCL10 levels are elevated in patients with various cardiovascular diseases, including coronary artery disease and acute myocardial infarction, and are associated with disease severity and inflammatory activity (16). Notably, sCD163 has been identified as a marker of macrophage activation that correlates with traditional cardiovascular risk factors, including age, HDL cholesterol, BMI, and Framingham risk score, suggesting its potential role in cardiovascular risk stratification (17). Furthermore, emerging evidence indicates that both sCD163 and CXCL10 exhibit distinct but complementary associations with cardiovascular disease risk factors and immune activation markers, supporting their combined utility in assessing disease progression (17). In patients with carotid atherosclerosis, the relationship between serum CXCL10 levels and systemic inflammatory cytokine profiles, as well as the distribution characteristics of peripheral blood immune cell subsets, remains inadequately understood. In particular, how the interplay between CXCL10 and anti-inflammatory pathways represented by sCD163 collectively influences the radiological progression of the disease lacks systematic clinical research evidence.

Accurately assessing disease activity and progression risk in patients with carotid atherosclerosis remains a significant challenge in clinical practice. While imaging techniques, such as carotid ultrasound, can provide direct visualization of plaque burden, degree of stenosis, and certain morphological features, they have limitations in reflecting the intrinsic immuno-inflammatory activity of plaques and predicting long-term progression. Consequently, identifying convenient, non-invasive serum biomarkers that can indicate the state of the immune microenvironment in carotid atherosclerosis and are closely associated with radiological progression has become a focal point of current research. To this end, the present study was designed as a clinical retrospective investigation. It aims to systematically examine the alteration patterns of serum sCD163 and CXCL10 levels in patients with carotid atherosclerosis and analyze their correlations with a panel of inflammatory cytokines and the distribution of key peripheral blood immune cell subsets. More importantly, by conducting 12-month follow-up carotid ultrasounds and categorizing patients into disease-stable and progression groups based on imaging changes, this study seeks to evaluate the predictive efficacy of baseline serum sCD163 and CXCL10 levels for radiological progression of carotid atherosclerosis. We anticipate that this research will contribute novel clinical data to the understanding of the roles of sCD163 and CXCL10 within the immunopathological network of carotid atherosclerosis. Furthermore, it will preliminarily assess the feasibility of using them as novel serological biomarkers for predicting disease progression, thereby offering a potential laboratory-based foundation for risk stratification and personalized management of carotid atherosclerosis.

## 2 Methods

### 2.1 Participants

**Figure 1:**
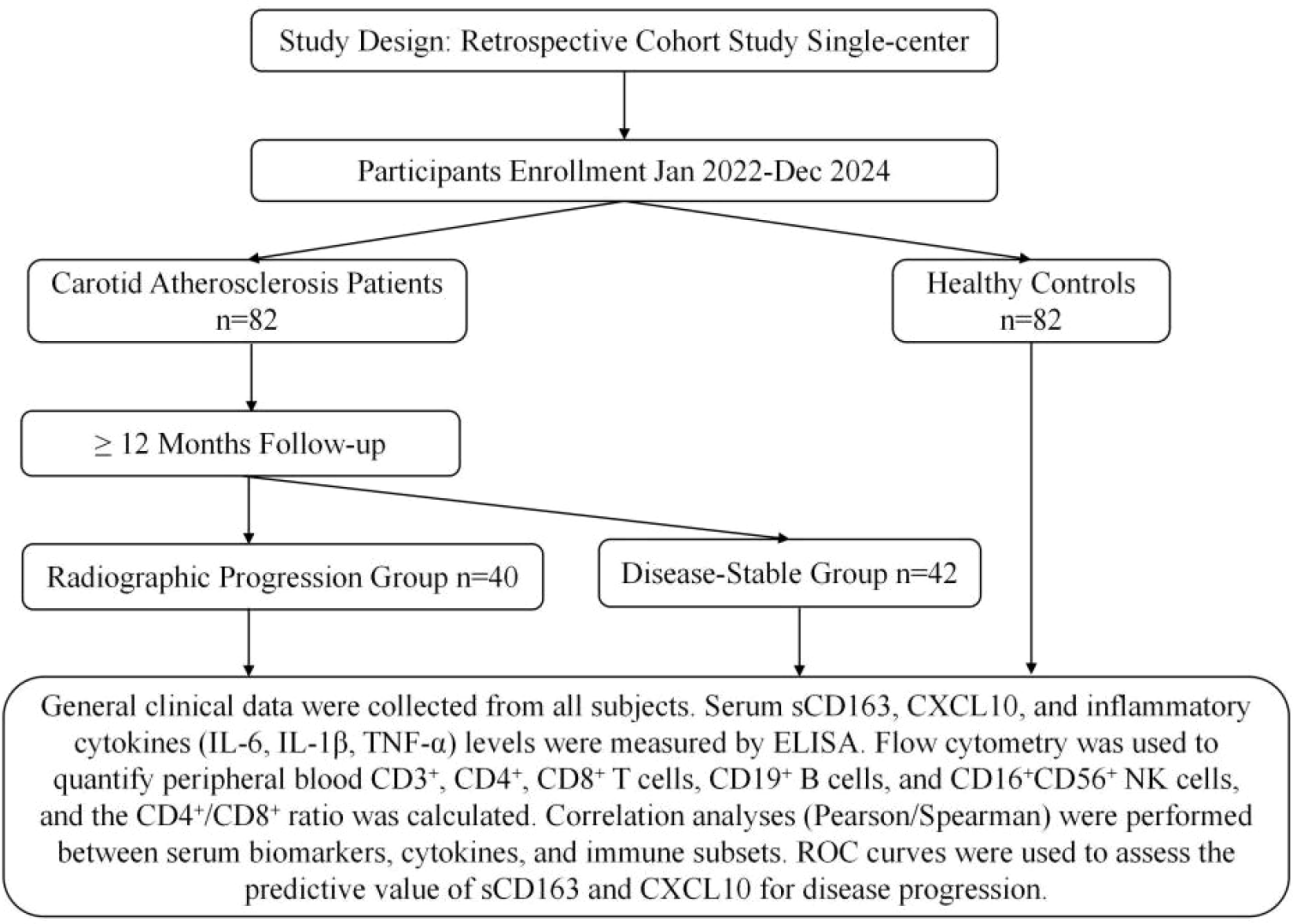
Study flow.

In this retrospective study, the case group comprised 82 patients diagnosed with carotid atherosclerosis via carotid color Doppler ultrasound from January 2022 to December 2024. Concurrently, eighty-two healthy adults who underwent physical examinations at the Health Management Center of our hospital during the same period, matched for age and sex, were selected as the healthy control group to assess the systemic immune-inflammatory characteristics of patients with carotid atherosclerosis. Peripheral blood samples were collected from all carotid atherosclerosis patients upon admission for laboratory index testing, and standardized carotid ultrasound examinations were performed. Subsequently, clinical records were reviewed to obtain follow−up ultrasound data at approximately 12 months from baseline. Since this was a retrospective study, patient selection and group assignment (stable vs. progression) were performed after the 12−month imaging outcomes were already known (post−hoc outcome−based grouping). Based on imaging changes, the carotid atherosclerosis patients were divided into a disease−stable group and a radiological progression group for inter−group comparative analysis. See **Figure 1**.

### 2.2 Inclusion criteria

1. Inclusion Criteria for Patients with Carotid Atherosclerosis: Aged 18–80 years, regardless of gender; diagnosed with carotid atherosclerotic plaque confirmed by carotid ultrasound, defined as: carotid intima-media thickness (IMT) ≥1.0 mm, or a localized IMT ≥1.5 mm protruding into the lumen, or the presence of a definite atherosclerotic plaque (18); complete clinical data available, including demographic characteristics, past medical history, medication history, and laboratory results; ability to complete at least 12 months of clinical follow-up.
2. Inclusion Criteria for Healthy Control Group: Aged 18–80 years, regardless of gender; carotid ultrasound shows no significant abnormalities (IMT <1.0 mm, no atherosclerotic plaque); no definite history of cardiovascular or cerebrovascular diseases; no active infection, autoimmune disease, or malignancy; no recent use of immunomodulatory drugs or hormonal medications; complete physical examination data and serum samples available.

### 2.3 Exclusion criteria

1. Exclusion Criteria for Patients with Carotid Atherosclerosis: Concurrent with other serious cardiovascular or cerebrovascular diseases, such as acute myocardial infarction, heart failure, severe arrhythmia, intracranial hemorrhage, or acute ischemic stroke; concurrent with known autoimmune diseases or active infectious diseases; concurrent with malignancy or hematologic disorders; concurrent with severe hepatic or renal insufficiency; use of medications affecting the immune system (e.g., glucocorticoids, immunosuppressants, or biologic agents) within the past 3 months; previous history of carotid endarterectomy or carotid artery stenting; pregnant or lactating women; poor-quality ultrasound images that preclude accurate assessment of plaque characteristics.
2. Exclusion Criteria for Healthy Control Group: Presence of carotid atherosclerotic plaque or IMT ≥1.0 mm; uncontrolled risk factors for atherosclerosis such as hypertension, diabetes, or hyperlipidemia; concurrent with acute/chronic infections, autoimmune diseases, or malignancy; use of medications affecting immune function within the past 3 months; pregnant or lactating women.

### 2.4 Ethical statement

This study protocol was approved by the Ethics Review Committee of our institution (approval no. WZY2025-KT-026-01) and conducted in accordance with the Declaration of Helsinki. As a retrospective analysis using anonymized residual samples, the ethics committee approved the waiver of written informed consent. All patient data were strictly protected to ensure privacy and confidentiality.

### 2.5 Sample size calculation

This study was designed as a retrospective, exploratory investigation. Given the absence of prior published data on the combined predictive value of sCD163 and CXCL10 for radiological progression of carotid atherosclerosis in the Chinese population, a formal *a priori* sample size calculation was not feasible. Instead, we adopted a pragmatic approach based on the available caseload at our institution during the study period (January 2022 to December 2024). A total of 82 eligible patients with carotid atherosclerosis and 82 age- and sex-matched healthy controls were enrolled.

To assess whether this sample size provided adequate statistical power for the primary analysis—ROC curve evaluation of the combined biomarker model—we conducted a *post-hoc* power analysis. Assuming an AUC of 0.75 for the null hypothesis (no discriminative ability) and an observed AUC of 0.913 for the combined model (from our results), with a 1:1 allocation between stable (n=42) and progression (n=40) groups, a two-sided type I error rate of 0.05, the achieved power exceeded 0.99, as calculated using PASS 2021 software (NCSS, LLC, USA). Thus, the sample size was adequate for the primary predictive analysis.

However, we acknowledge that the comparison between stable and progression groups was defined *post-hoc*, and the subgroup sample sizes were not pre-specified. Therefore, findings related to between-group comparisons should be interpreted as exploratory and hypothesis-generating. External validation in larger, prospective cohorts is warranted.

### 2.6 Treatment and data collection

All patients with carotid atherosclerosis received standardized secondary prevention treatment according to current clinical guidelines, including antiplatelet therapy, statin lipid-lowering therapy, blood pressure management, and blood glucose control. Medication regimens were determined by treating physicians independent of this study, and baseline medication use was recorded for all patients (**Table 1**). Importantly, there were no significant between-group differences in the use of statins, antiplatelet agents, antihypertensive, or glucose-lowering medications, ensuring comparability of pharmacological background between the stable and progression groups.

**Table 1:** Baseline demographic and clinical characteristics.

Indicators	Healthy control group (n=82)	Disease-stable group (n=42)	Radiographic progression group (n=40)	Statistic	*P*-value
Demographic Characteristics					
Age (years, mean ± SD)	59.65 ± 9.27	61.33 ± 8.69	62.12 ± 9.50	*F* = 1.121^1^	0.328
Gender, n (%)				χ^2^ = 0.132^2^	0.936
Male	48 (58.54)	26 (61.90)	24 (60.00)		
Female	34 (41.46)	16 (38.10)	16 (40.00)		
BMI (kg/m², mean ± SD)	23.64 ± 2.28	23.51 ± 2.55	23.88 ± 2.81	*F* = 0.235^1^	0.791
Cardiovascular Risk Factors					
Smoking History [n (%)]	18 (21.95)	14 (33.33)	16 (40.00)	χ^2^ = 4.681^2^	0.096
Hypertension [n (%)]	—	28 (66.67)	30 (75.00)	χ^2^ = 0.687^2^	0.407
Diabetes Mellitus [n (%)]	—	12 (28.57)	15 (37.50)	χ^2^ = 0.740^2^	0.390
Coronary Heart Disease [n (%)]	—	8 (19.05)	10 (25.00)	χ^2^ = 0.424^2^	0.515
Laboratory Examinations					
TC (mmol/L, mean ± SD)	4.32 ± 0.87	4.79 ± 0.92^#^	4.89 ± 1.03^#^	*F* = 6.764^1^	0.002
TG (mmol/L, median (Q1, Q3))	1.57 (1.19, 1.99)	1.68 (1.23, 2.13)	1.86 (1.54, 2.51)^#^	H=10.999^3^	0.004
LDL-C (mmol/L, mean ± SD)	2.69 ± 0.52	3.29 ± 0.85^#^	3.69 ± 0.94^#,&^	*F* = 27.262^1^	<0.001
HDL-C (mmol/L, mean ± SD)	1.48 ± 0.22	1.19 ± 0.20^#^	1.01 ± 0.17^#,&^	*F* = 77.881^1^	<0.001
FPG (mmol/L, mean ± SD)	5.23 ± 0.68	6.12 ± 1.45^#^	6.88 ± 2.03^#,&^	*F* = 22.089^1^	<0.001
HbA1c (%, mean ± SD)	5.42 ± 0.38	6.23 ± 1.12^#^	7.01 ± 1.45^#,&^	*F* = 39.252^1^	<0.001
Medication Use					
Standard Statin Therapy [n (%)]	—	38 (90.48)	32 (80.00)	χ^2^ = 1.800^2^	0.180
Antiplatelet Agents [n (%)]	—	36 (85.71)	32 (80.00)	χ^2^ = 0.473^2^	0.492
Antihypertensive Agents [n (%)]	—	26 (61.90)	28 (70.00)	χ^2^ = 0.597^2^	0.440
Glucose-lowering Agents [n (%)]	—	10 (23.81)	16 (40.00)	χ^2^ = 2.480^2^	0.115

^1^ One Way ANOVA; ^2^Pearson χ^2^ test; ^3^Kruskal-Wallis H test. #indicates *P*<0.01 compared with the healthy control group; & indicates *P* < 0.01 compared with the disease-stable group. The same as below.

### 2.7 Laboratory and imaging assessments

1. Fasting venous blood samples were collected from all participants into serum separator tubes. The samples were allowed to clot for 30 minutes at room temperature and then centrifuged at 1,500×g for 10 minutes. Serum aliquots were immediately separated and stored at −80 °C until further analysis. Serum levels of sCD163, CXCL10, and inflammatory cytokines (IL-6, IL-1β, TNF-α) were measured using commercial ELISA kits (R&D Systems, USA) according to the manufacturer’s instructions. All measurements were performed in duplicate, and the mean values were used for statistical analysis. ①sCD163 ELISA (R&D Systems, Cat# DC1630): detection range 62.5-4,000 pg/mL; sensitivity 31.3 pg/mL. ②CXCL10 ELISA (R&D Systems, Cat# DIP100): detection range 15.6-1,000 pg/mL; sensitivity 7.2 pg/mL. ③IL-6 ELISA (R&D Systems, Cat# D6050): detection range 3.1–300 pg/mL; sensitivity 0.7 pg/mL. ④IL-1β ELISA (R&D Systems, Cat# DLB50): detection range 3.9–250 pg/mL; sensitivity 1.0 pg/mL. ⑤TNF-α ELISA (R&D Systems, Cat# DTA00D): detection range 15.6-1,000 pg/mL; sensitivity 5.5 pg/mL.
2. Flow cytometry analysis of peripheral blood immune cell subsets: ①Sample collection and processing: Peripheral venous blood samples (5 mL) were collected into EDTA-anticoagulated tubes (BD Biosciences, USA) at baseline. All samples were processed within 4 hours of collection using a lyse/no-wash protocol to preserve cell integrity and minimize activation artifacts. ②Antibody staining: The following fluorochrome-conjugated monoclonal antibodies (all from BD Biosciences, USA) were used: anti-CD3 FITC (clone SK7), anti-CD4 PE (clone SK3), anti-CD8 PerCP-Cy5.5 (clone SK1), anti-CD19 APC (clone HIB19), and anti-CD16/CD56 PE-Cy7 (clone B73.1/NCAM16.2). Fifty microliters of well-mixed whole blood were incubated with the optimized antibody cocktail for 20 minutes at room temperature in the dark. Red blood cells were lysed using 450 μL of FACS Lysing Solution (BD Biosciences, USA) for 15 minutes at room temperature. Samples were then washed once with phosphate-buffered saline (PBS), centrifuged at 300×g for 5 minutes, and resuspended in 300 μL of PBS for immediate acquisition. ③Acquisition and gating strategy: Flow cytometric acquisition was performed on a BD FACS Canto II flow cytometer (BD Biosciences, USA) equipped with 488 nm and 633 nm lasers. For each sample, at least 50,000 events within the lymphocyte gate were acquired. The sequential gating strategy was as follows: Step 1: Lymphocytes were identified by their characteristic low forward scatter (FSC-A) and low side scatter (SSC-A) properties, excluding debris and aggregates. Step 2: Doublet discrimination was performed using FSC-A vs. FSC-H to exclude cell doublets, retaining only single cells. Step 3: CD3^+^ T cells were identified from the single-cell lymphocyte gate based on CD3 expression vs. SSC-A. Step 4: From the CD3^+^ gate, CD4^+^CD8⁻ and CD4⁻CD8^+^ populations were identified to determine CD4^+^ and CD8^+^ T cell percentages, respectively. CD4^+^CD8^+^ double-positive and CD4⁻CD8⁻ double-negative populations were excluded from the final subset counts. Step 5: CD19^+^ B cells were identified from the lymphocyte gate based on CD19 expression vs. SSC-A. Step 6: CD16^+^CD56^+^ NK cells were identified from the lymphocyte gate based on CD16/CD56 expression vs. SSC-A.
3. Biochemical parameters including total cholesterol (TC), triglycerides (TG), low-density lipoprotein cholesterol (LDL-C), high-density lipoprotein cholesterol (HDL-C), fasting plasma glucose (FPG), and glycated hemoglobin (HbA1c) were measured using an automatic biochemical analyzer (Beckman Coulter, AU5800, USA) following standard laboratory protocols.
4. Imaging Evaluation and Grouping: The assessment and follow-up of carotid atherosclerosis were performed using the same model of color Doppler ultrasound system (Philips EPIQ 7C). Measured parameters included: maximum plaque thickness (MPT), plaque area, carotid intima-media thickness (CIMT), and stenosis rate (based on blood flow velocity parameters). Radiological progression was defined as any of the following changes from baseline after the 12-month follow-up: an increase in MPT of any known plaque by ≥0.5 mm; the appearance of a new plaque; or an increase in stenosis degree by ≥50%. The ≥50% stenosis progression criterion, although stringent, was adopted based on prior studies evaluating significant atherosclerotic progression (19, 20). We acknowledge that this threshold may not capture more subtle but clinically relevant progression. Therefore, the present definition should be considered as one of several possible progression metrics. Based on the 12-month follow-up results, patients with carotid atherosclerosis were categorized into a disease-stable group (not meeting the above progression criteria) and a radiological progression group. To assess inter-observer and intra-observer variability, all ultrasound examinations were independently reviewed by two experienced radiologists who were blinded to clinical and biomarker data. A third senior radiologist resolved any discrepancies. Intraclass correlation coefficients (ICCs) for MPT and CIMT measurements were 0.94 and 0.92, respectively, indicating excellent reproducibility.

### 2.8 Statistical analysis

Statistical analyses were performed with SPSS 26.0. Normally distributed continuous data are presented as mean ± SD and compared via one-way ANOVA; non-normal data as median (Q1-Q3) with the Kruskal-Wallis H test. Categorical data are shown as n (%) and analyzed using the chi-square or Fisher’s exact test. For comparisons involving three groups (healthy control, disease-stable, and radiological progression) in **Tables 1**–**3**, *post-hoc* pairwise comparisons were adjusted using the Bonferroni correction, with a corrected significance threshold of α’ = 0.05/3 = 0.0167. Comparisons between only two groups (e.g., baseline characteristics between stable and progression groups in **Table 1**, and ROC analysis in **Table 4**) did not require Bonferroni adjustment. Correlations were assessed with Pearson or Spearman tests. The predictive value of baseline sCD163 and CXCL10 for 12−month radiological progression was evaluated by ROC curve analysis, reporting AUC, cutoff, sensitivity, and specificity. Two−sided P < 0.05 was considered significant except where Bonferroni correction was applied (α’ = 0.0167).

**Table 2:** Comparison of serum sCD163, CXCL10 levels and inflammatory cytokine levels across groups (pg/mL).

Indicators	Healthy control group (n=82)	Disease-stable group (n=42)	Radiographic progression group (n=40)	Statistic	*P*-value
sCD163	152.65 ± 37.45	185.72 ± 59.41^#^	236.88 ± 52.31^#,&^	*F* = 42.261^3^	<0.001
CXCL10	186.81 ± 43.41	245.62 ± 61.53^#^	358.91 ± 72.57^#,&^	*F* = 124.926^3^	<0.001
IL-6	3.48 (2.81, 4.46)	5.97 (4.86, 7.58)^#^	12.18 (9.47, 13.83)^#,&^	H=91.408^3^	<0.001
IL-1β	0.79 (0.57, 1.09)	1.56 (1.03, 2.01)^#^	3.19 (2.41, 4.29)^#,&^	H=98.931^3^	<0.001
TNF-α	6.30 (4.60, 7.62)	9.20 (7.62, 12.15)^#^	17.92 (14.67, 22.42)^#,&^	H=100.966^3^	<0.001

**Table 3:** Comparison of immune cell subset distribution across groups (mean ± SD).

Indicators	Healthy control group (n=82)	Disease-stable group (n=42)	Radiographic progression group (n=40)	Statistic	*P*-value
CD3^+^ T Cells (%)	69.32 ± 3.46	68.73 ± 3.71	67.55 ± 3.77^#^	*F* = 3.243^1^	0.042
CD4^+^ T Cells (%)	41.51 ± 10.14	44.57 ± 12.92	46.44 ± 9.29	*F* = 3.120^1^	0.047
CD8^+^ T Cells (%)	26.68 ± 3.73	23.80 ± 4.33^#^	21.10 ± 2.86^#,&^	*F* = 31.612^1^	<0.001
CD4^+^/CD8^+^ Ratio	1.55 ± 0.30	1.86 ± 0.40^#^	2.21 ± 0.36^#,&^	*F* = 50.044^1^	<0.001
CD19^+^ B Cells (%)	12.16 ± 2.41	12.04 ± 2.05	13.00 ± 2.02	*F* = 2.377^1^	0.096
CD16^+^CD56^+^ NK Cells (%)	16.94 ± 3.81	17.46 ± 2.96	18.16 ± 4.58	*F* = 1.387^1^	0.253

**Table 4:** ROC curve analysis for serum sCD163 and CXCL10 in predicting radiological progression of carotid atherosclerosis.

Variables	CXCL10	sCD163	Joint prediction
AUC	0.875	0.767	0.913
95% CI	0.783-0.938	0.660-0.853	0.830-0.964
Youden Index	0.679	0.493	0.711
Optimal Cut-off Value	>320.12	>189.03	—
Sensitivity (%)	75.00	85.00	92.00
Specificity (%)	92.86	64.29	78.57
Z-value	9.520	5.042	12.688
*P*-value	<0.001	<0.001	<0.001

## 3 Results

### 3.1 Baseline characteristics

**Table 1** compares baseline characteristics among a healthy control group, a stable carotid atherosclerosis group, and a progression group. No significant intergroup differences were found in demographics, or cardiovascular risk factors (*P*>0.05). However, both patient groups had higher TC, LDL-C, FPG, and HbA1c, and lower HDL-C than controls (*P*<0.01). Furthermore, the progression group had significantly higher LDL-C, FPG, HbA1c, and TG, and lower HDL-C than the stable group (*P* < 0.01). Notably, medication use was well-balanced between the disease-stable and radiological progression groups, with no significant differences in standard statin therapy, antiplatelet agents, antihypertensive agents, or glucose-lowering agents (all P>0.05). This comparability minimizes potential confounding by pharmacological treatment on subsequent biomarker analyses.

### 3.2 Comparison of serum sCD163, CXCL10 levels and inflammatory cytokine levels across groups

**Table 2** compares the serum levels and serum inflammatory cytokine levels among the healthy control group, the disease-stable group, and the radiological progression group. The results indicate a graded increase in serum sCD163 and CXCL10 levels across the three groups: they were lowest in the healthy control group, intermediate in the disease-stable group, and highest in the radiological progression group (*P* < 0.001). Specifically, the levels of both sCD163 and CXCL10 in the progression group were significantly higher than those in the stable group (*P* < 0.001). IL-6, IL-1β, and TNF-α were significantly higher in the progression group than in both the stable and control groups (*P* < 0.001), and levels in the stable group were also significantly higher than in controls (*P* < 0.001).

### 3.3 Comparison of immune cell subset distribution across groups

**Table 3** presents differences in peripheral blood immune cell subsets among healthy controls, stable carotid atherosclerosis patients, and progression patients. Compared with controls, both patient groups showed significantly altered immune cell proportions: CD3^+^ T cells were lower in the progression group (*P*<0.01), CD8^+^ T cells were reduced in both patient groups and lowest in the progression group (*P* < 0.001), and the CD4^+^/CD8^+^ ratio was elevated, highest in the progression group (*P* < 0.001).

### 3.4 Correlation analysis of serum sCD163, CXCL10, inflammatory cytokines and immune cell subsets

**Figure 2:**
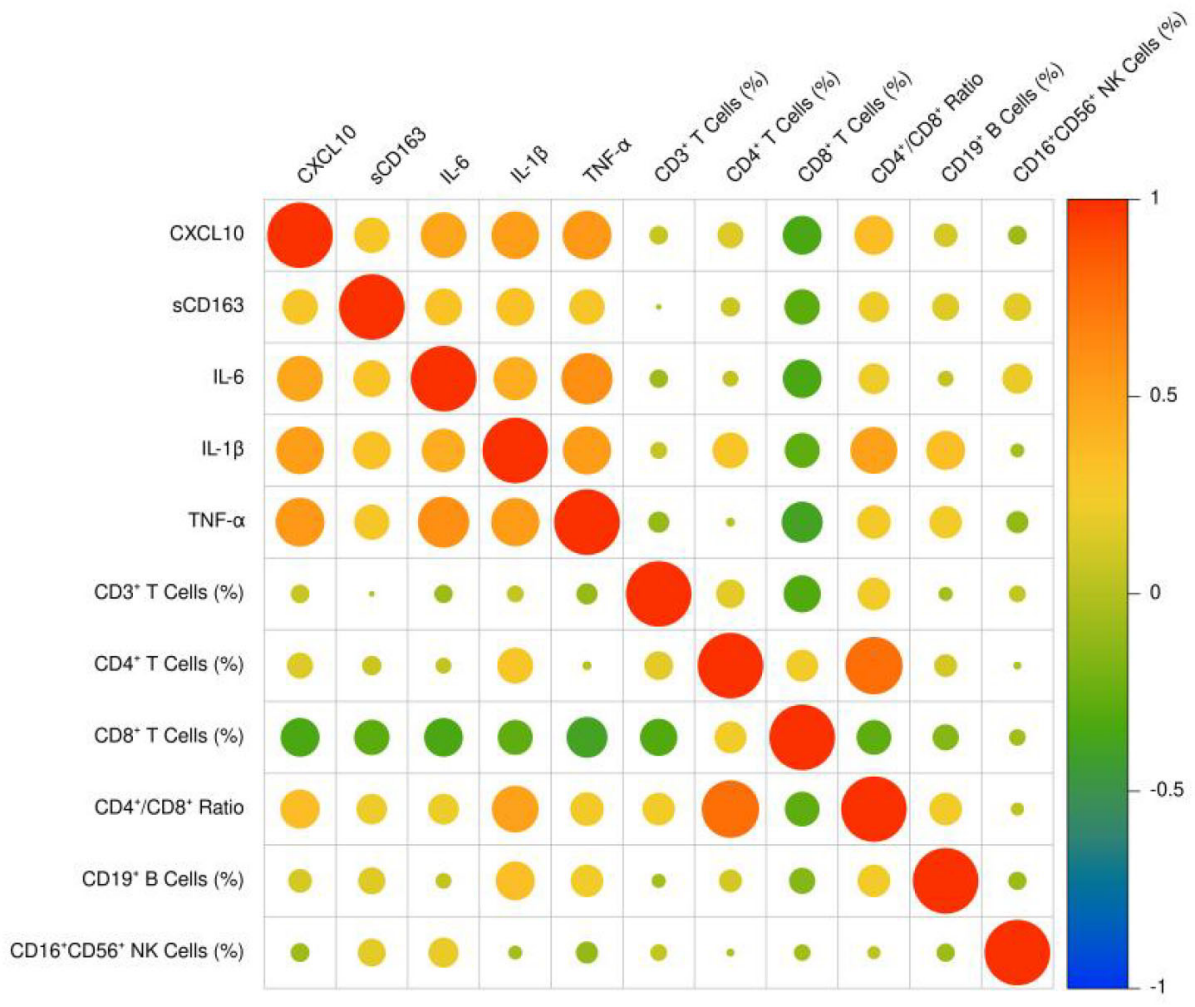
Heatmap for the correlation analysis of serum sCD163, CXCL10, inflammatory cytokines, and immune cell subsets.

**Table 5** and **Figure 2** presents the correlation analysis between serum levels of sCD163 and CXCL10 and both inflammatory cytokines and immune cell subsets in patients with carotid atherosclerosis. Spearman’s correlation analysis revealed that CXCL10 was moderately to strongly positively correlated with IL-6 (ρ=0.523), IL-1β (ρ=0.572), and TNF-α (ρ=0.570), while sCD163 showed weaker but still significant positive correlations with these cytokines (ρ=0.376, 0.385, and 0.346, respectively; all P<0.01). Regarding immune cell subsets, neither CXCL10 nor sCD163 was significantly correlated with the percentages of CD3^+^ T cells, CD4^+^ T cells, CD19^+^ B cells, or CD16^+^CD56^+^ NK cells. However, CXCL10 was significantly negatively correlated with the percentage of CD8^+^ T cells (r=-0.344, P = 0.002) and positively correlated with the CD4^+^/CD8^+^ ratio (r=0.352, P = 0.001). Similarly, sCD163 also demonstrated a significant negative correlation with CD8^+^ T cell percentage (r=-0.284, P = 0.010) and a positive correlation with the CD4^+^/CD8^+^ ratio (r=0.211, P = 0.047). These findings suggest that elevated levels of sCD163 and CXCL10 are associated with a systemic pro-inflammatory state and an imbalance in T-cell homeostasis, characterized by reduced CD8^+^ T cells and an increased CD4^+^/CD8^+^ ratio, in patients with progressive carotid atherosclerosis.

**Table 5:** Correlation analysis of serum sCD163, CXCL10, inflammatory cytokines and immune cell subsets.

Indicators	CXCL10		sCD163	
	*r/ρ-*value	*P*-value	*r/ρ-*value	*P*-value
IL-6	*ρ* = 0.523	<0.001	*ρ* = 0.376	0.001
IL-1β	*ρ* = 0.572	<0.001	*ρ* = 0.385	<0.001
TNF-α	*ρ* = 0.570	<0.001	*ρ* = 0.346	<0.001
CD3^+^ T Cells (%)	*r=*-0.076	0.499	*r=*0.006	0.959
CD4^+^ T Cells (%)	*r=*0.152	0.172	*r* = 0.083	0.461
CD8^+^ T Cells (%)	*r=*-0.344	0.002	*r=*-0.284	0.010
CD4^+^/CD8^+^ Ratio	*r* = 0.352	0.001	*r* = 0.211	0.047
CD19^+^ B Cells (%)	*r* = 0.125	0.264	*r* = 0.165	0.138
CD16^+^CD56^+^ NK Cells (%)	*r* = -0.077	0.490	*r* = 0.173	0.120

### 3.5 Predictive efficacy of serum sCD163 and CXCL10 for radiological progression

**Table 4**, **Figure 3** show ROC curve results for predicting 12-month radiological progression in carotid atherosclerosis using baseline CXCL10 and sCD163, individually and combined. CXCL10 yielded an AUC of 0.875 (95% CI: 0.783-0.938), cut-off >320.12 pg/mL, sensitivity 75.00%, specificity 92.86%. sCD163 showed an AUC of 0.767 (95% CI: 0.660-0.853), cut-off >189.03 pg/mL, sensitivity 85.00%, specificity 64.29%. The combination improved performance (AUC: 0.913; 95% CI: 0.830-0.964; sensitivity 92.00%, specificity 78.57%), suggesting CXCL10 and sCD163 together may serve as a promising biomarker pair for identifying high-risk patients.

**Figure 3:**
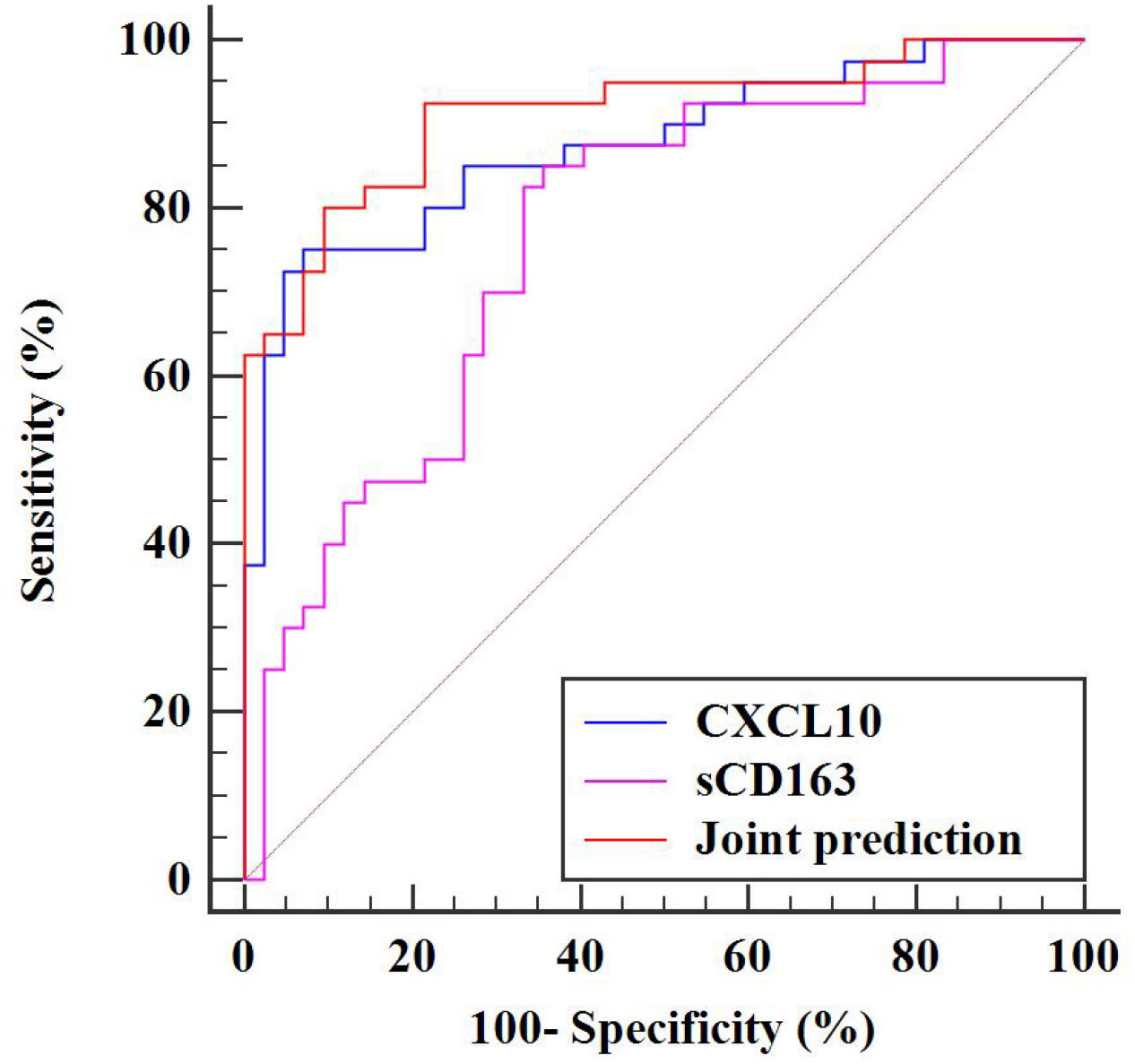
ROC curves of serum CXCL10, sCD163, and combined prediction for predicting radiological progression of carotid atherosclerosis.

## 4 Discussion

Carotid atherosclerosis is a chronic immuno-inflammatory disease rather than simple lipid deposition (21, 22). Innate and adaptive immune cells shape the plaque’s local immune microenvironment, critically influencing its progression and rupture risk (23). This study revealed that patients with radiological progression had significantly higher serum sCD163 and CXCL10 levels than stable patients, and both markers correlated with inflammatory cytokines and immune cell subsets. These findings suggest that sCD163 and CXCL10 are associated with carotid atherosclerosis progression at the systemic level. However, whether these molecules directly drive intraplaque pathogenesis requires further histological and mechanistic validation.

Vulnerable plaques are characterized by a large necrotic core, inflammation, and impaired tissue repair (24). Macrophages are functionally heterogeneous immune cells within plaques. CD163+ macrophages (the Mhem/M(Hb) subtype), due to their expression of the scavenger receptor for the hemoglobin-haptoglobin complex, were once considered to have anti-atherosclerotic effects, such as secreting the anti-inflammatory cytokine IL-10 and promoting reverse cholesterol transport (25–27). However, recent studies have shown that CD163-deficient mice develop smaller and less complex plaques, suggesting a potential pro-atherosclerotic role. Furthermore, previous small-sample studies on CD163+ macrophages and plaque stability have yielded conflicting results (28). Soluble CD163 (sCD163), the soluble form of the hemoglobin-haptoglobin complex scavenger receptor CD163, has long been a focus in atherosclerosis research. Through a large-scale histological study of 200 human carotid artery plaques, Bengtsson et al. (29) were the first to demonstrate, at both protein and mRNA levels, a positive correlation between CD163 expression and plaque vulnerability, challenging the traditional view that CD163+ macrophages solely confer protection against atherosclerosis. Their study found that CD163 protein expression was significantly higher in plaques from patients with cerebrovascular symptoms (*P* < 0.001) and showed a significant positive correlation with the plaque vulnerability index (*P* < 0.001). CD163+ macrophages were primarily localized in the plaque shoulder region, an anatomical feature highly consistent with areas at high risk for plaque rupture. In our study, the significantly elevated sCD163 levels in the progression group are consistent with the histological findings of Bengtsson et al. (29), who reported increased CD163 expression in vulnerable plaques. However, because we did not perform intraplaque analysis, we cannot claim that circulating sCD163 directly reflects intraplaque CD163^+^ macrophage activation. Our data demonstrate a systemic association; direct histological validation is required to establish such a relationship. Furthermore, in our study, sCD163 was positively correlated with IL-6, IL-1β, and TNF-α (*r* = 0.355, 0.287, 0.294, all *P* < 0.001), further supporting the clinical value of sCD163 as a systemic inflammation marker. Its elevation may indicate an active inflammatory response and tissue damage repair process within the plaque. The study by Li et al. (30) provides prospective evidence linking circulating sCD163 levels with clinical prognosis. That study enrolled 290 patients with carotid atherosclerosis and 46 non-carotid atherosclerosis controls. After a 24-month follow-up, baseline plasma CD163 levels were significantly higher in patients who experienced MACE compared to those who did not (P = 0.033). After multivariate adjustment, each unit increase in CD163 was associated with a 41.3% increased risk of MACE (HR = 1.413, 95% CI: 1.022-1.954, *P* = 0.036). This finding importantly complements the results of our study, where sCD163 predicted radiological progression (AUC = 0.767). The study by Li et al. (30) established the predictive value of sCD163 for clinical endpoints (stroke, myocardial infarction, death), while our study further confirms its predictive efficacy for subclinical disease progression (radiological progression).

CXCL10 is a key chemokine mediating Th1-type immune responses. The study by Gencer et al. (31) indicated that CXCL10, expressed by T cells and monocytes, promotes T cell retention within lesions via its receptor CXCR3 and mediates the homing of activated Th1 cells to sites of plaque development. Saigusa et al. (32) further confirmed that both Th1 cells and CD8^+^ T cells highly express CXCR3 in human atherosclerotic plaques, and plasma CXCL10 levels are significantly elevated in patients with coronary atherosclerosis. In the present study, the correlation of CXCL10 with inflammatory cytokines was significantly stronger than that of sCD163. Furthermore, CXCL10 correlated significantly with both a lower percentage of CD8^+^ T cells and a higher CD4^+^/CD8^+^ ratio in peripheral blood. These systemic associations suggest a potential link between CXCL10 and adaptive immune regulation, but intraplaque validation—such as immunohistochemical analysis of CXCL10 and CXCR3 expression within carotid plaques—is necessary to confirm a local role. These characteristics of immune imbalance align with the more active inflammatory state observed in the progression group patients. The decrease in CD8^+^ T cells observed in this study may reflect the migration and infiltration of activated cytotoxic T cells into the local plaque site, rather than systemic T cell exhaustion. In terms of predictive efficacy, CXCL10 outperformed sCD163 (AUC 0.875 vs. 0.767). Its optimal cutoff value (320.12 pg/mL) corresponded to a specificity as high as 92.86%, suggesting that CXCL10 may be a more precise biomarker for identifying patients at high risk of progression. This finding is consistent with the trend of upregulated CXCL10 expression in plaques and aligns with its biological property as an IFN-γ-induced product. Prapiadou et al. (33) used integrated proteogenomic and Mendelian randomization analyses to confirm CXCL10 as a potential downstream causal mediator of IL-6 signaling in atherosclerosis. Reduced CXCL10 levels mediated up to 67% of the effect of genetically downregulated IL-6 signaling on cardiovascular outcomes. Additionally, high CXCL10 expression was linked to a larger lipid core in human carotid plaques and transcriptomic signatures of immune cell infiltration and adaptive immune activation. Furthermore, the study by Segers et al. (34) indicated that CXCL10 expression levels in human carotid endarterectomy specimens were closely associated with plaque morphological features and stability. The strong correlation between CXCL10 and IL-6 (r=0.517) further supports its close link to plaque inflammatory activity. Notably, the study by Castley et al. (17) showed that although both sCD163 and CXCL10 are associated with monocyte/macrophage activation and cardiovascular risk, they exhibit distinct association patterns with HIV clinical parameters and cardiovascular disease risk factors, suggesting that CXCL10 may more specifically reflect T cell-mediated adaptive immune responses.

To contextualize our findings, baseline sCD163 levels in our healthy control group (152.65 ± 37.45 pg/mL) were comparable to those reported in previous studies of healthy populations (approximately 140–180 pg/mL) (35, 36). However, our progression group showed markedly elevated levels (236.88 ± 52.31 pg/mL), which exceed values typically observed in stable coronary artery disease (190–210 pg/mL) but are lower than those in acute myocardial infarction (250–300 pg/mL) (37). This positioning suggests that progressive carotid atherosclerosis represents an intermediate inflammatory state between stable chronic disease and acute cardiovascular events. Similarly, CXCL10 levels in our progression group (358.91 ± 72.57 pg/mL) were substantially higher than those in healthy controls (typically 100–200 pg/mL) and exceeded values reported in stable coronary artery disease (200–280 pg/mL) (16).

A potential concern is that pharmacological interventions, particularly statins, may influence circulating levels of sCD163, CXCL10, and inflammatory cytokines. However, several considerations support the validity of our findings. First, medication use was comparable between the stable and progression groups (**Table 1**), ensuring that inter-group comparisons reflect disease-related rather than treatment-related differences. Second, any anti-inflammatory effect of standard therapy would be expected to affect both groups similarly, preserving the relative differences observed. Third, the persistence of significantly elevated biomarkers in the progression group despite comparable medication use suggests that sCD163 and CXCL10 capture residual inflammatory activity insufficiently suppressed by standard secondary prevention. This is clinically relevant, as these biomarkers may identify patients requiring intensified therapeutic strategies. Future prospective studies should evaluate the dynamic response of these biomarkers to specific pharmacological interventions.

This study systematically revealed the synergistic role of serum sCD163 and CXCL10 in the progression of carotid atherosclerosis and their association with systemic immune-inflammatory status in a Chinese population. Analysis based on ROC curves demonstrated that the combined detection of these two markers had high predictive efficacy for disease progression (AUC = 0.913). This provides clinicians with a non-invasive and convenient serological biomarker panel, holding promise for the early identification of carotid atherosclerosis patients at high risk of progression. Building upon traditional imaging assessments, dynamic monitoring of sCD163 and CXCL10 can enable a more comprehensive evaluation of plaque inflammatory activity and immune microenvironment status. This offers a new laboratory basis for formulating individualized treatment strategies, evaluating therapeutic efficacy, and guiding secondary prevention.

Beyond sCD163 and CXCL10, other inflammatory molecules have also been shown to accumulate in atherosclerotic lesions and promote plaque progression via macrophage activation and leukocyte recruitment. For instance, previous studies have demonstrated that various chemokines and cytokines contribute to the complex network of inflammatory signals that drive atherosclerosis (38, 39). Our findings do not imply that sCD163 and CXCL10 are unique or sufficient drivers of disease progression; rather, they represent two measurable components of a broader systemic inflammatory milieu. Future studies integrating multiplex biomarker panels with intraplaque histological analyses will be necessary to dissect the relative contributions of individual inflammatory mediators.

## 5 Study limitations

As a single−center retrospective study, this research had a relatively limited sample size, and all patients were recruited from a single medical center, potentially introducing selection bias and limiting the generalizability of the findings. Importantly, because patients were selected retrospectively after their 12−month radiological outcomes were known (post−hoc outcome−based grouping), the study design is inherently susceptible to selection bias and cannot establish a true temporal predictive relationship. This is a major limitation that must be considered when interpreting the reported predictive performance of sCD163 and CXCL10. Furthermore, the 12-month follow-up period, while sufficient to observe radiological progression, is relatively short, and the predictive value for clinical endpoints needs confirmation through longer-term follow-up. This study did not include histological analysis of plaques, precluding direct verification of the association between serum markers and local plaque CD163^+^ macrophage infiltration or CXCL10 expression. Moreover, the observational design precludes the establishment of causality. Although sCD163 and CXCL10 showed significant associations with disease progression, their causal roles in disease pathogenesis and progression require further validation through animal models and intervention studies. Due to the retrospective design, longitudinal serum samples at the 12-month follow-up were not available, and therefore, we were unable to assess dynamic changes in sCD163, CXCL10, or inflammatory cytokines over time. This limitation precludes evaluation of whether biomarker trajectories parallel disease progression and represents an important direction for future prospective studies. Furthermore, because follow-up blood samples were not available, we could not evaluate longitudinal changes in T cell subsets or their potential modulatory effects on cytokine dynamics during disease progression. The cross-sectional nature of our immune cell analysis limits causal inference regarding the relationship between immune cell shifts and biomarker elevation. Additionally, detailed information on dietary habits, lifestyle factors (including physical activity, sleep quality, and psychological stress), and use of over-the-counter anti-inflammatory medications or nutritional supplements was not collected in this retrospective study. These factors are known to influence systemic inflammatory cytokine profiles and may have confounded the observed associations between sCD163, CXCL10, and disease progression. Future prospective studies should incorporate structured assessments of diet, lifestyle behaviors, and comprehensive medication histories to better control for these potential confounders.

## 6 Conclusion

In summary, this study confirmed that serum levels of sCD163 and CXCL10 are closely associated with the radiological progression of carotid atherosclerosis, and their combined detection exhibits good predictive efficacy. These two markers are associated with systemic inflammatory status and peripheral immune cell imbalance, suggesting a coordinated involvement of innate and adaptive immunity in disease progression. However, direct validation of intraplaque biology was not performed in this study. sCD163 and CXCL10 hold potential as novel biomarkers for risk stratification and individualized management of carotid atherosclerosis. Future research should focus on validating their predictive value for clinical endpoints and exploring their application scenarios in therapeutic decision-making and treatment efficacy monitoring, thereby advancing the development of precision medicine for carotid atherosclerosis.

## Data Availability

The original contributions presented in the study are included in the article/supplementary material. Further inquiries can be directed to the corresponding author.
